# The Effect of Daily Practice of Puzzle-Game Apps on Cognition in Two Groups of Older Adults: A Pre-Post Experimental Study

**DOI:** 10.3390/ijerph192315454

**Published:** 2022-11-22

**Authors:** Noa Givon Schaham, Zvi Buckman, Debbie Rand

**Affiliations:** 1 Department of Occupational Therapy, Sackler Faculty of Medicine, Tel Aviv University, Tel Aviv 6997801, Israel; 2 Maccabi-Healthcare Services, Rishon L’Zion 7526602, Israel

**Keywords:** mild cognitive impairment, subjective cognitive decline, cognitive training, touchscreen tablet

## Abstract

There is an urgent need for non-pharmacological cognitive interventions to delay the onset and modify the progression of the cognitive deterioration of older adults with early stages of cognitive decline. ‘Tablet Enhancement of Cognition and Health’ (TECH) is such an intervention. We aimed to assess the suitability of TECH for older adults with and without mild cognitive impairment (MCI). Specifically, we wanted to explore the feasibility and to determine the initial effectiveness of TECH for older adults with Pre-Mild Cognitive Impairment (pre-MCI) as well as with MCI. This is pre-post experimental design, including two groups of older adults. Feasibility included group session attendance (adherence), self-training time (compliance), and satisfaction from the TECH intervention. The Montreal Cognitive Assessment (MoCA) assessed global cognition and the WebNeuro computerized battery assessed specific cognitive components. Twenty-eight participants with MCI (8 women, aged 65–87), and ten participants with pre-MCI (5 women, aged 65–86) participated in TECH. High adherence, compliance, and satisfaction were reported by both groups. Memory recall improved for the MCI group (z = −2.7 *p* = 0.006). In addition, for the MoCA an intermediate effect size (Cohen’s d = 0.52) and a small effect (Cohen’s d = 0.18) were found for the MCI and pre-MCI groups, respectively. Large to small effect size values for WebNeuro cognitive components were found for both groups. Both groups of older adults were motivated, performed daily self-training, which gave them enjoyment and a sense of control. TECH seems to have potential to preserve cognition over time. Additional research with a longer follow-up is needed to determine whether TECH can prevent cognitive decline in older adults with MCI but especially with pre-MCI.

## 1. Introduction

The pace of population ageing is much faster than in the past. Countries worldwide face major challenges to ensure that their health and social systems are ready for this demographic shift [1]; neurocognitive frailty is one of the biggest threats to successful aging [2]. Age-related cognitive decline (rather than physical decline) is also the biggest fear of older adults [3]. Therefore, many older adults are seeking ways to preserve their cognitive status and prevent future decline [4].

In a range between healthy cognition and dementia, there is a continuum of cognitive decline, which causes concern for many older adults. The main syndrome in this continuum is Mild cognitive impairment (MCI), defined as cognitive decline greater than expected for an individual’s age and education level, but that does not notably interfere with performing activities of daily life [5]. Impaired memory and executive function deficits are common at this stage [5]. Executive functions are higher cognitive abilities such as working memory and cognitive flexibility. Older adults with pre-MCI perceive subtle cognitive changes that are not yet detected with clinical tests, therefore have a subjective feeling of cognitive decline [6,7], which may be one of the earliest noticeable symptoms of MCI [8].

The United Nations General Assembly declared 2021–2030 as the Decade of Healthy Ageing [1]. Non-pharmacological cognitive interventions can promote healthy aging by delaying the onset and the progression of cognitive deterioration [9] in older adults with early stages of cognitive decline. In recent years, there has been growing interest in training programs designed to improve cognitive abilities in older adults, particularly for those with cognitive deficits [10]. Dozens of studies have investigated training programs using technology, such as computer software or video-games, and have shown to have potential for improving cognitive functions in older adults with no cognitive decline [11] as well as for older adults with MCI, with some interventions showing moderate to large effect sizes [12,13,14]. In 2020 Lampit et al. updated the most comprehensive review regarding the effectiveness of computerized cognitive training in cognitively healthy older adults. It included a total of 90 trials, and showed a small but statistically significant effect on global cognitive functioning favoring computerized cognitive training over the control group [15]. However, it is not clear if the benefits from computerized cognitive training programs are transferred to improve everyday cognitive functioning [11,14].

The ‘Tablet Enhancement of Cognition and Health’ (TECH) is a novel occupational therapy intervention developed for older adults with MCI, and includes daily self-training using touchscreen tablet puzzle-game apps, facilitated by weekly group sessions. TECH aims to prevent decline of global cognition as well as of different cognitive components such as memory and executive functions via an ongoing cognitive leisure activity.

Learning how to operate the touchscreen tablet, to use every-day functional apps (such as a camera, news sites, YouTube, etc.), and to practice puzzle-game apps, are all activities that facilitate the learning of new cognitive skills. Indeed, participation in cognitive leisure activities, which incorporates the learning of new cognitive skills, has been found to be associated with a reduced risk of developing dementia [16]. Participating in cognitive leisure activities using computer platforms may enhance such benefits, as a result of the increased cognitive stimulation they provide [17].

Reducing the risk of dementia is also associated with the frequency of practice (participation in such activities). Cognitive training several times a week, which was achieved with TECH, has been found to reduce the risk of dementia by 50% more than training once a week [16].

The feasibility of TECH, as well as its effectiveness to preserve global cognition, has been established for older adults with MCI [18], but not for older adults with pre-MCI. Therefore, we aimed to determine whether TECH is feasible to use with older adults with pre-MCI as well as our cohort with MCI and within each group to assess changes in cognitive and executive functions between pre and post intervention with TECH.

We hypothesized that TECH would be feasible for older adults with MCI as well as for older adults with pre-MCI and initial effectiveness will be found for both groups of older adults.

## 2. Materials and Methods

### 2.1. Study Design

This Pre-post experimental design included two groups of older adults; with MCI and pre-MCI. This is a secondary analysis of two studies: the experimental arm of a randomized control trial (clinical trial number NCT02955277) and a pre-post experimental design (clinical trial number NCT02955303). Both groups of older adults participated in the TECH intervention. Assessments pre and post the TECH intervention were conducted by assessors who were blind to group allocation (MCI group) or not involved in the intervention (pre-MCI group). Assessors were not aware who belonged to the MCI or pre-MCI group.

### 2.2. Population

Community-dwelling older adults (>65 years) were referred to the study by their family or geriatric physician. Additional inclusion criteria: independence in activities of daily living (as verified by self-report regarding Basic Activities of Daily Living (BADL) and the Lawton Instrumental Activities of Daily Living (IADL) Scale [19]), subjective memory complaints that were supported by a family member, having normal or corrected vision and hearing, written and spoken fluency of language, the ability to use a touchscreen tablet after an initial demonstration, and not having severe depressive symptoms [Geriatric Depression Scale (GDS) [20] > 10], and other neurological or psychiatric conditions. Individuals with a score of 19–25/30 points in the Montreal Cognitive Assessment (MoCA) [21], a valid and reliable tool for cognitive status, were included in the MCI group and individuals with a MoCA score of 25 and above, indicating they had subjective cognitive decline were included in the pre-MCI group. All participants signed informed consent before participating in the study.

### 2.3. Tools

#### 2.3.1. Tools to Describe the Population

The Lawton Instrumental Activities of Daily Living (IADL) Scale [19] is a reliable and valid self-report questionnaire, which consists of eight IADL items. The total score ranges from 0 (completely dependent) to 23 (completely independent). This questionnaire was used to verify that participants perform all of the daily tasks they have always performed.

The Geriatric Depression Scale (GDS) [20] is a valid and reliable self-rating screening tool developed to detect depressive symptoms in older adults. The questionnaire includes 15 yes/no statements. Score above 10 points indicates the presence of depressive symptoms.

#### 2.3.2. Outcome Measures

Feasibility testing of TECH intervention included Adherence—attendance in the six weekly group sessions, Compliance—self-training hours per week, and total training hours—from participants’ daily logs and their iPad ‘Screen Time’ app information. In addition, the names of the apps that were played were recorded. Satisfaction from the intervention was rated by the participants after TECH completion using a questionnaire. This questionnaire [18] included 10 questions rated on a 1–5 Likert scale (e.g., How much did the self-training motivate you to make an effort?), and 5 questions on a 1–3 Likert scale (e.g., Was the TECH intervention too long/just the right length/too short?).

Global cognition was assessed using MoCA [21], which was also used to screen for eligibility. The MoCA is a valid and reliable cognitive screening tool with high sensitivity and specificity, aimed to distinguish individuals with MCI from healthy adults. The MoCA assesses attention and concentration, executive functions, memory, language, visuo-constructional skills, conceptual thinking, calculations, and orientation; the scores range from 0 to 30. Higher score indicates better cognition, the norms for individuals with MCI range between 19–25 points, score of 26–30 means no clinical detected cognitive changes [21].

Specific cognitive components (such as executive functions and memory) were assessed using the WebNeuro assessment tool [22]. This is a valid neuropsychological computerized assessment battery including the assessment of the following specific cognitive components (subtests): Sustained attention (Continuous Performance Task), controlled attention (Verbal Interference Task), flexibility (Switching of Attention Task), inhibition (Go-NoGo Task), working memory (Digit Span Task), memory recall (Memory Recall Task), and problem solving (Maze Task). For each component the software calculated a raw score that was then converted to a z score, with a normative average of 0, and a standard deviation of 1. For each subtest, higher scores indicate better performance. In addition, demographic information and previous technology experience (e.g., computer, smartphone, and tablet) were collected.

#### 2.3.3. The TECH Intervention

All participants received the TECH intervention, which included daily self-training facilitated by weekly group sessions. Participants received iPads to take home and were requested to play puzzle-game apps 3–5 times a week X 30–60 min, for a total of 15–25 training sessions. The weekly one-hour sessions took place in a small group setting and were led by an occupational therapist. The group sessions included the following: teaching tablet operation as well as allowing participants to explore and practice new apps to increase their self-confidence and independence in using the tablet. TECH utilized a variety of apps in terms of complexity and interest to address individual participant’s cognitive level and treatment needs. For the self-training sessions, the occupational therapist selected several apps for each participant to play independently at home. Because apps were not specifically developed for cognitive rehabilitation, they required the use and integration of different EF components (and not isolated components), which facilitated practicing different cognitive components, such as working memory, problem solving, and reasoning. From the options selected, participants could choose what apps to use at home. At later stages, participants were also encouraged to independently search for additional apps that interested them.

### 2.4. Procedure

Older adults were approached by phone and were provided with information about the study. Individuals who were willing to participate were invited to the geriatric clinic for the assessment session. After signing an informed consent form, the MoCA, GDS, Basic, and Instrumental Activities of Daily Living (BADL and IADL) questionnaires were administered to confirm eligibility and to form the MCI and pre-MCI groups. All participants received the TECH intervention, which was carried out in several rounds including 4–6 participants. Each small group included participants with MCI as well as participants with pre-MCI, without distinguishing between them. In the first group session, participants were given a tablet and an illustrated manual to take home for self-training.

### 2.5. Data Analysis

Data were imputed to ‘IBM SPSS Statistics 25′ software. Owing to the small and uneven groups, and since the MoCA and WebNeuro scores, pre and post intervention, were not normally distributed (Shapiro–Wilk Test), non-parametric statistics were used. 

Descriptive statistics [median (inter quartile range—IQR)] were used to characterize the sample and the feasibility of using the TECH intervention (adherence, compliance, and satisfaction). For each of the two groups the differences between the pre and post intervention in global cognition and cognitive components were tested using the Wilcoxon test. In addition, the percent change for each of the variables was calculated using this formula [(post-pre)/pre X 100%]. When the percentage change indicated improvement, Cohen’s d effect size, which indicates the magnitude of change, was also calculated. First, Cohen’s r effect size values, for non-parametric tests, were calculated by using the following formula [Cohen’s r = Z/√N]. Then, the values were converted to Cohen’s d [23]. Cohen’s d effect size values were considered small (>0.1), intermediate (>0.4), and large (>0.7) [24]. Effect size indicates clinical meaningfulness, which goes beyond statistical significance, which is also highly dependent on the sample size. 

In each of the groups, the percentage of participants who achieved the MoCA’s Minimal Clinically Important Difference (MCID) was also calculated. MCID is considered the smallest change in scores perceived by the patient as beneficial [25]. Since the MCID for the MoCA has not yet been established in older adults with MCI, in this study, we relied on the MoCA MCID established for older adults during stroke rehabilitation, which showed an improvement of at least 1.22 points [26]. The MCID for the WebNeuro battery has also not yet been established.

## 3. Results

Since this is a secondary analysis our two groups were not equal in size; 28 participants with MCI [who were assigned to receive TECH in the RCT (8 women and 14 men aged 65–87 (mean age 76.3)], and 10 participants with pre-MCI [5 women and 5 men aged 65–86 (mean age 72.4)]. During the intervention, three participants from the MCI group dropped out [due to a decline in their health condition (N = 1) and lack of interest (N = 2)]; therefore, the feasibility data was collected from the remaining 25 participants. There were no dropouts in the pre-MCI group. Twenty-three of the 25 participants from the MCI group filled out the satisfaction questionnaire, and 22 of them reported their self-training time. All ten participants from the pre-MCI group filled out the satisfaction questionnaire, and nine of them reported their self-training hours. 

As per the inclusion criteria, all participants were independent in BADL and IADL and were without severe depressive symptoms. Most participants reported using a smartphone and/or a computer on a daily basis prior to the study. Previous touchscreen tablet experience was reported by most of the pre-MCI participants but only by a few of the MCI participants, which was found to be statistically significant (z = 7.7, *p* < 0.001). No between-group differences were found for self-efficacy as well as for most of the other demographic characteristics (see Table 1).

**Figure 1:**
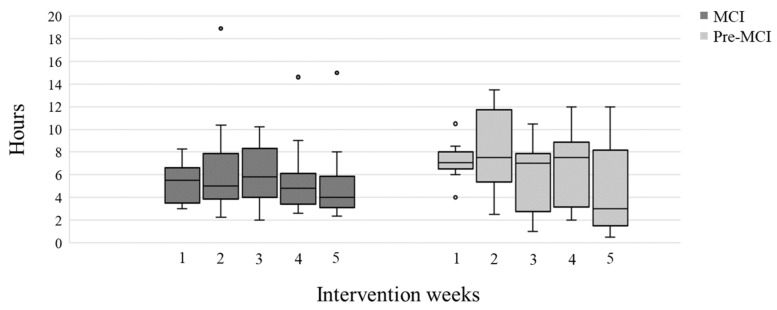
Box-plots of self-training hours for each of the 5 weeks of intervention, for the 22 participants with MCI (dark grey) and the 9 participants with pre-MCI (light grey). Colored circles represent potential outliers; participants with extreme self-training time.

Participants from both groups attended at least 80% of the six group sessions. The total self-training time for the MCI group ranged from 5.3 to 50.1 h, with a median (IQR) total training time of 23.62 (16.9–29.1) hours, 4.7 (3.8–5.8) hours per week. The total self-training time for the pre-MCI group was higher, ranging from 12.5 to 53.5 h, with a median (IQR) total training time of 31.7 (18.9–40.9) hours during the 5-week intervention, 6.3 (3.8–8.2) hours per week; however, this was not statistically significant (U = 70.5, *p* > 0.05). The training time for both groups was consistent throughout the five-week intervention (Figure 1).

Very high satisfaction from the TECH intervention was reported by 75% of the MCI participants and 90% of the pre-MCI participants. The participants reported that the intervention motivated them to a great extent to make an effort (93.7% of the MCI participants and 70% of the pre-MCI participants) and they were highly satisfied with the option to self-train, specifically regarding the cognitive stimulation during self-training (75% of the MCI participants and 100% of the pre-MCI participants). Table 2 presents the ratings of the satisfaction questionnaire.

The median (IQR) percentage of MoCA change from pre to post intervention for the MCI participants was 4.2 [(−4.2)–11.3] and 1.7 [(−5.5)–4.6] for the pre-MCI participants. These changes were not statistically significant (see Table 3). However, an intermediate effect size was found for the MCI group (Cohen’s d = 0.52) and a small effect (Cohen’s d = 0.18) for the pre-MCI group. Post intervention, 48% of the MCI participants and 20% of the pre-MCI participants achieved an improvement in MoCA MCID. Four participants (16%) with MCI had a post MoCA score of at least 1.22 points lower than their pre score, and the MoCA score of one of them declined below the MCI definition. In the pre-MCI group, two participants (20%) at post intervention had a MoCA score of less than 1.22 points from the pre intervention, and they matched the MCI definition.

Table 3 presents the WebNeuro cognitive and executive function scores pre and post the TECH intervention and the percentage change for both groups. The following improvements were found for the MCI group: a large effect size (Cohen’s d > 1.0) with statistical significance (Z = −2.7 *p* = 0.006) for Memory Recall, an intermediate effect size for Controlled Attention (Cohen’s d = 0.45), and a small effect size for the Total Thinking Score (Cohen’s d = 0.12). In the pre-MCI group, the following improvements were found: an intermediate effect size for Working Memory (Cohen’s d = 0.57) and the WebNeuro Total Thinking Score (Cohen’s d = 0.43); a small effect size for Inhibition (Cohen’s d = 0.23) and Flexibility (Cohen’s d = 0.11).

## 4. Discussion

TECH, which includes intensive cognitive stimulation while learning and practicing a new cognitive skill of tablet use [18,27], provides a leisure activity for older adults. Here, we combined groups from two previous studies to show the effect of TECH participation of older adults with MCI as well as older adults with pre-MCI. TECH was found to be highly feasible for older adults with MCI, as well as for older adults with pre-MCI, as reflected by their high adherence and compliance with the intervention. Both groups reported very high satisfaction with the intervention, especially with the self-training component of TECH.

High self-training time was found for older adults with MCI, which was surprisingly even higher for older adults with pre-MCI. Participants with pre-MCI, due to their enhanced cognitive abilities, possibly perceived greater success in playing the different puzzle-game apps and therefore played for longer periods of time without taking breaks. This success also might have been translated into a higher motivation, which led to additional practicing. Perhaps these participants were also aware of the possible positive benefits to their cognitive performance, which supports previous research of older adults who used gaming apps on a regular basis and reported that the apps improved their cognitive functioning [28].

Differences between groups were also found for the variety and type of the apps used during the self-training sessions. Participants with pre-MCI used puzzle-game apps, which are cognitively more complex and require the use of higher cognitive abilities, such as problem solving and reasoning (such as ‘Move the Box’ app). Conversely, participants with MCI used easier puzzle-games apps, which are less cognitively challenging (such as the ‘Flow Free’ app). This observation is interesting because, although the apps were selected by the occupational therapists, participants at home were free to choose what apps they which to engage in. It is unclear whether selecting more challenging apps for participants with MCI could have led to higher cognitive practice, requiring more effort, or whether this would have led to frustration and consequently decreased their motivation to practice. These findings provide us with a deeper understanding regarding the older adult’s functional level and their need for cognitive stimulation. Further research is needed.

Participants reported high satisfaction with the intervention and a great desire to continue practicing beyond the requested length of time. This was achieved by utilizing this exciting and novel technology of touchscreen tablets, which motivated them to practice [29,30]. Older adults have reported high satisfaction from touchscreen tablet use in clinical settings for different purposes (including two studies specifically for cognitive training) [30,31]. Some of the TECH participants reported to struggle with stop using the app, since they were having such a positive experience. The addictive features of using gaming apps with older adults have been reported previously; older adults exhibited heavier use patterns compared with younger groups [28], perhaps since they have fewer responsibilities or more leisure time.

Positive effect size values (without statistical significance) were found for global cognition (the MCI group) and for different cognitive components (both groups). The effect size calculation indicates the clinical meaningfulness of the intervention despite not reaching statistical significance, due to the small sample size [32]. These findings support previous research. A study using tablet-training methods for older adults [30] found small effect size values for different cognitive components, with significant improvement only for processing speed. No significant improvement in specific cognitive components was reported in a recent systematic review and meta-analysis [30], and the effect size was not calculated.

Different patterns of self-training were observed between groups for participants that decreased in global cognition. Participants with MCI who decreased their global cognition trained less than the group median training time, but participants with pre-MCI trained for a longer time (compared with the pre-MCI group median training time). Possibly, participants with pre-MCI, who were aware of their initial clinical cognitive decline, had increased motivation for training. This insight reinforces the importance of maintaining involvement in cognitive leisure activities, such as TECH at early stages. Improving occupational engagement, such as participating in cognitive leisure activities, may promote health and well-being of older adults with subjective cognitive decline, and may delay future cognitive and functional decline [4]. For example, older adults that participated leisure activities with high cognitive demands such as quilting and digital photography showed enhanced memory functions [33]. These results were also found for older adults who learned how to use a tablet device, providing them also a new technological skill, which was useful in facilitating everyday activities such as banking [34].

We need to acknowledge a few limitations: this study is a secondary analysis and therefore might lack sufficient power. Groups were not equal in size and are relatively small, especially the pre-MCI group, which might have affected our ability to detect statistically significant changes. Follow-up assessments could have been helpful in showing the effect of TECH over time. Because not all participants completed the self-training logs and satisfaction questionnaire, it makes it difficult to draw broader conclusions on these issues. Participants were highly educated; thus findings might not generalize to older adults with less years of education.

## 5. Conclusions

TECH intervention motivated older adults with MCI and pre-MCI to perform daily self-training, which gave them enjoyment while participating in cognitive leisure activities. TECH seems to have potential to preserve cognition over time. Additional research with a longer follow-up period is needed to determine whether TECH can prevent cognitive decline in older adults with MCI and especially for pre-MCI.

## Figures and Tables

**Table 1:** Demographic characteristics of the participants in both groups.

		MCI (N = 30)	Pre-MCI (N = 10)	Independent Samples *t*-Test
		Mean ± SD Min-Max	Mean (SD) Min-Max	t (*p*)
Age (years)		76.3 WQ± 5.3 65–87	72.4 ± 6.9 65–86	1.8 (0.07)
Education (years)		13.6 ± 4.1 8–30	16.3 ± 2.5 12–20	−1.9 (0.06)
MoCA (0–30)		22.7 ± 1.9 19–25	27.3 ± 0.9 26–29	−7.4 (<0.001)
GSES (10–40)		33.6 ± 4.5 19–39	31.2 ± 6.5 19–38	1.2 (0.2)
IADL Questionnaire (0–23)		21.8 ± 2.9 8–23	23.1 ± 0.3 23–24	−1.3 (0.2)
		N (%)	N (%)	χ2 (*p*)
Sex	Female	13 (46.4)	5 (50)	0.04 (0.85)
	Male	15 (53.6)	5 (50)	
Residence	Alone	5 (17.9)	4 (40)	5.3 (0.07)
	With family	23 (82.1)	6 (60)	
Main occupation	Working	6 (21.4)	3 (30)	0.3 (0.6)
	Retired	22 (78.6)	7 (70)	
Drive	Yes	23 (82.1)	10 (100)	2.0 (0.1)
Computer use	Yes	23 (82.1)	10 (100)	5.1 (0.2)
Smartphone use	Yes	24 (85.7)	10 (100)	1.6 (0.2)
Tablet use	Yes	6 (21.4)	7 (70)	7.7 (0.005)

**Table 2:** Satisfaction Questionnaire regarding participation in TECH. The grey lines represent the questions answered.

	**MCI** **(N = 23)**	**Pre-MCI** **(N = 10)**
	**%**	**%**
General satisfaction from TECH		
Very Satisfied	47.8	60.0
Satisfied	30.4	30.0
Neutral	21.7	10.0
Satisfaction from the group sessions		
Very Satisfied	28.6	70.0
Satisfied	50.0	30.0
Neutral	13.6	
Enjoyment from the group sessions		
Enjoyed very much	50	50.0
Enjoyed	31.8	30.0
So-so	18.2	20.0
Satisfaction from self-training at home		
Very Satisfied	52.2	60.0
Satisfied	34.8	30.0
Neutral	13.0	10.0
Satisfaction from persisting to self-train over-time		
Very Satisfied	17.4	30.0
Satisfied	47.8	40.0
Neutral	30.4	20.0
Slightly Satisfied	4.3	10.0
Motivation from the self-training		
Very motivated	39.1	30.0
Motivated	47.8	40.0
Neutral	13.0	30.0
Satisfaction from the cognitive demands		
Very Satisfied	39.1	50.0
Satisfied	43.5	50.0
Neutral	13.0	
Slightly Satisfied	4.3	
Perceived improvement following TECH		
Substantial improvement	13.0	11.1
Some improvement	30.4	33.3
Neutral	39.1	22.2
Very little improvement	8.7	22.2
Not at all	8.7	11.1
The demand for daily training		
Too short	26.1	22.2
Just right	47.8	55.6
Too long	26.1	22.2
Duration of the program		
Too short	69.6	50.0
Just Right	26.1	50.0
Too long	4.3	
Will you continue to practice following TECH?		
Yes	86.4	85.7
No	13.6	14.3

**Table 3:** **a.** The Median (IQR) pre and post scores, the percentage change, and the pre-post differences for the **MCI group**. **b**. The Median (IQR) pre and post scores, the percentage change, and the pre-post differences for the pre-MCI group.

(a)					
		MCI (N = 25)			
		Pre	Post	% Change Pre-Post	Differences Between Pre-Post
		Median IQR	Median IQR	Median IQR	Z(*p*)
MoCA (0–30)		23.0 21.0–24.0	23.0 21.5–25.0	4.2 −4.2–11.3	−1.2 (0.2)
WebNeuro Computerized Cognitive Battery	Sustained Attention	−0.6 −1.0–(−0.1)	−0.5 −0.9–0.2	−35.2 −109.1–61.4	−0.2 (0.8)
	Controlled Attention	−1.2 −1.7–(−0.6)	−1.1 −1.5–(−0.9)	9.0 −21.6–66.1	−1.1 (0.3)
	Flexibility	−1.2 −1.9–(−0.5)	−1.2 −1.9–(−0.7)	−0.1 −42.6–20.5	−0.4 (0.7)
	Inhibition	−0.3 −0.8−0.2	0.0 −0.6–0.4	−53.1 −157.3–85.3	−0.7 (0.4)
	Working Memory	−1.4 −1.9–(−0.8)	−1.1 −1.8–(−0.8)	−14.9 −51.6–21.9	−1.3 (0.2)
	Memory Recall	−0.9 −1.8–0.1	−1.2 −2.2–(−0.3)	1.9 −84.1–126.6	−2.7 (0.006)
	Problem solving	0.2 −0.3–0.6	0.5 −0.2–0.8	−12.9 −148.1–66.0	−0.5 (0.6)
	Total Thinking Score	−0.6 −1.1–(−0.3)	−0.7 −0.9–(−0.3)	0.5 −38.8–41.3	−0.3 (0.8)
**(b)**					
		**Pre-MCI (N = 10)**			
		**Pre**	**Post**	**% Change** **Pre-Post**	**Differences Between Pre-Post**
		**Median** **IQR**	**Median** **IQR**	**Median** **IQR**	**Z(*p*)**
MoCA (0–30)		27.0 26.75–28.0	28.0 26.0–29.0	1.7 −5.5–4.6	−0.3 (0.8)
WebNeuro Computerized Cognitive Battery	Sustained Attention	−0.04 −0.5–0.6	0.1 −0.3–0.4	−19.0 −88.3–143.3	−0.8 (0.4)
	Controlled Attention	−0.5 −1.1–(−0.3)	-0.05 −0.9–(−0.1)	−23.6 −59.8–77.5	−1.1 (0.2)
	Flexibility	−0.2 −0.9–0.7	−0.3 −1.9–0.8	10.5 −16.3–113.5	−0.2 (0.8)
	Inhibition	0.2 −0.3–0.5	0.2 −0.7–0.6	0.0 −16.7–159.0	−0.3 (0.7)
	Working Memory	−1.1 −1.9–0.2	−1.2 −1.8–(−0.6)	6.0 −26.3–22.0	−0.9 (0.4)
	Memory Recall	0.1 −0.5–0.6	−0.0 −0.8–0.6	−6.4 −124.3–161.7	−1.0 (0.3)
	Problem solving	0.1 −0.2–0.6	0.6 −0.1–0.8	−0.8 −261.1–335.7	−1.8 (0.06)
	Total Thinking Score	−3 −0.4–0.1	−0.2 −0.5–0.3	23.2 −25.6–106.8	−0.6 (0.5)

## Data Availability

The data presented in this study are available on request from the corresponding author. The data are not publicly available due to the Helsinki restriction.
